# Malic Acid for the Treatment of Rheumatic Diseases

**DOI:** 10.31138/mjr.301223.mar

**Published:** 2023-12-30

**Authors:** Jozélio Freire de Carvalho, Aaron Lerner

**Affiliations:** 1 Núcleo de Pesquisa em Doenças Crônicas não Transmissíveis (NUPEN), School of Nutrition from the Federal University of Bahia, Salvador, Bahia, Brazil,; 2 Chaim Sheba Medical Centre, The Zabludowicz Research Centre for Autoimmune Diseases, Israel,; 3 Ariel University, Ariel, Israel

**Keywords:** rheumatic diseases, rheumatoid arthritis, malic acid, inflammation

Malic acid (MA) is an intermediate compound of tricarboxylic acid cycle (TCA) and its addition could increase energy production. There are few articles demonstrating efficacy with low toxicity of MA supplementation in fibromyalgia (FM).^1,2^ Therefore, in this aspect, a systematic review of all articles published that evaluated the role of MA supplementation in rheumatic disease was presently conducted.

Following PRISMA guidelines, an extensive literature search in PubMed, Scielo, and LILACS was performed without any language restriction, from 1965 to March 2023. After a review of titles and abstracts, only three articles were selected for this review.

Those three articles are summarised in **Table 1.**^1,3,4^ The articles evaluated the effects of MA in Sjogren’s syndrome (SS) and FM with a total of 114 patients (190 SS and 24 in FM). Female patients were dominated, and ages varied from 56.17 to 58.5 years. All three studies were randomised controlled trials. In SS, MA was given as stimulatory saliva tapioca drug and in FM oral capsules were given to the patients. All trials showed an improvement in the clinical pictures, including the salivary flow and improvement of xerostomia symptoms in SS; and a decrease in FM symptoms was noted. No side effects were identified.

**Table 1.** Studies on malic acid in rheumatic diseases.

**Author, year**	**Study design**	**Country**	**Age, gender**	**N**	**Disease**	**D-ribose dosage**	**Outcome**	**Side effects**
Da Silva Marques et al., 2011^3^	Double-blind randomised controlled trial	Portugal	56.17 yo, 100% females	80	Sjögren’s syndrome	(Xeros) (malic acid 4.33% w⁄ w), and compare it with a traditional citric acidadded GSSS (SST) (Sinclair Pharma Plc, Godalming, UK) (malic acid 4.2% and citric acid 2.1% w⁄ w)	Both GSSS significantly stimulated salivary output without significant differences. The new gustatory stimulant of salivary secretion presented an absolute risk reduction of 52.78% [33.42–72.13 (95% CI)] when compared with the traditional one.	ND
Da Mata et al., 2019^4^	Randomized controlled cross-over trial	Portugal	58.5 yo, 98.5% females	110	Sjögren’s syndrome	Acid malic lozenge (4.33% w⁄w) from Xeros® system) and citric acid mouthwash were used four times a day for 2 weeks	Greater effect size and percentage improvement than citric acid mouthwash. The malic acid lozenge also produced a significant greater salivary output	ND
Russell et al., 1995^1^	Randomised, double-blind, placebo controlled, crossover pilot study	United States	ND	24	Fibromyalgia	200mg plus magnesium 50mg	With dose escalation and a longer duration of treatment in the open label trial, significant reductions in the severity of all 3 primary pain/tenderness measures were obtained.	None

FM: fibromyalgia; ND: not described; yo: years.

There are some other articles in the literature showing efficacy of topical MA in patients with xerostomia. In fact, a metanalysis on this field including 5 articles with 244 patients with xerostomia receiving topical sialagogue spray (malic acid 1%) or placebo (5). The authors evaluated and analysed weather topical sialagogue spray (malic acid 1%) improved the symptoms of dry mouth significantly better than the placebo, evaluated by the following questionnaires: Dry Mouth Questionnaire (DMQ), Xerostomia Inventory (XI), and Visual Analogue Scale (VAS) scores. Furthermore, an increase in unstimulated and stimulated saliva flow rates was also seen in treated SS patients applying MA.^4^ The suggested mechanism of action of MA, to improve salivary flow, is that after using a spray containing malic acid 1%, the hydrogen ion (H+) dissociates from malic acid to bind water (H_2_O), generating hydronium ions (H_3_O+). Then, H_3_O+ stimulates the secretion of saliva from salivary glands to neutralise acid, thereby improving dry mouth.^5^

The present study’s strengths are: 1. the inclusion of all studies on MA in rheumatic conditions; 2. the use of international criteria for rheumatic diseases; 3. an extensive, long-term, and multilingual literature search was performed. The limitations are the detection of only few studies. Therefore, future well designed studies using MA in rheumatic conditions are desired to confirm the present data and expand on other rheumatic diseases. In conclusion, only a few studies evaluated the role of malic acid in rheumatic diseases like SS and FM. Future well designed, using MA in rheumatic conditions, are highly desired to confirm the present data and expand on other rheumatic diseases.
